# Liquid Flow and Mass Transfer Behaviors in a Butterfly-Shaped Microreactor

**DOI:** 10.3390/mi12080883

**Published:** 2021-07-27

**Authors:** Haicheng Lv, Zhirong Yang, Jing Zhang, Gang Qian, Xuezhi Duan, Zhongming Shu, Xinggui Zhou

**Affiliations:** State Key Laboratory of Chemical Engineering, East China University of Science and Technology, Shanghai 200237, China; HchenLv@163.com (H.L.); carlqg@ecust.edu.cn (G.Q.); xzduan@ecust.edu.cn (X.D.); zmshu@ecust.edu.cn (Z.S.); xgzhou@ecust.edu.cn (X.Z.)

**Keywords:** millimeter-scale, computational fluid dynamics (CFD), droplet, liquid-liquid flow, flow pattern and mass transfer

## Abstract

Based on the split-and-recombine principle, a millimeter-scale butterfly-shaped microreactor was designed and fabricated through femtosecond laser micromachining. The velocity fields, streamlines and pressure fields of the single-phase flow in the microreactor were obtained by a computational fluid dynamics simulation, and the influence of flow rates on the homogeneous mixing efficiency was quantified by the mixing index. The flow behaviors in the microreactor were investigated using water and n-butanol, from which schematic diagrams of various flow patterns were given and a flow pattern map was established for regulating the flow behavior via controlling the flow rates of the two-phase flow. Furthermore, effects of the two-phase flow rates on the droplet flow behavior (droplet number, droplet size and standard deviation) in the microreactor were investigated. In addition, the interfacial mass transfer behaviors of liquid–liquid flow were evaluated using the standard low interfacial tension system of “n-butanol/succinic acid/water”, where the dependence between the flow pattern and mass transfer was discussed. The empirical relationship between the volumetric mass transfer coefficient and Reynold number was established with prediction error less than 20%.

## 1. Introduction

Since the 1990s, microreactor technology has been widely used in the fields of pharmaceuticals, emulsion preparation, and chemical process enhancement [1,2,3]. Compared with conventional industrial reactors, microreactors have smaller feature sizes and larger specific surface areas, thereby enabling efficient mass and heat transfer and highly controlled and continuous operation of the process [4]. Excellent mixing performance is one of the major advantages of microreactors [5]. However, the flow in microreactors is often laminar with a low Reynold number, where the mixing is mainly driven by molecular diffusion. Many studies have been carried out to control or improve the mixing performance by employing external sources of energy such as electric fields [6], magnetic fields [7], ultrasonic fields [8], etc., which are often costly and difficult to integrate. Therefore, most of the current research is based on changing the structure of microchannels to achieve effective fluid contact and mixing.

For passive mixing processes, there are a number of structural design strategies to enhance the mixing. The very basic design strategy is to use curved structures, such as serpentine [9] and zigzag [10], to create chaotic flows to facilitate transverse mass transfer. There are also design ideas that take advantage of the Coanda effect, such as the Tesla structure [11], to form or increase the transverse dispersion. In addition, there is a way of repeatedly stretching and shearing the fluid through the structure of split and recombine [12,13,14]. For the split and recombine structure, previous research [15] illustrated that the asymmetric structure can effectively improve the mixing effect.

For the abovementioned microreactors, the channel size is usually in the submillimeter scale, and the small characteristic diffusion distance facilitates the mixing efficiency. However, for reactions which not only require a certain degree of mixing, but also a sufficient residence time, such as nitration [16,17] and hydrolysis [18], it may not be desirable to use these submillimeter-sized reactors due to their limited liquid-holding capacities. At present, studies of flow patterns within the millimeter-scale microreactors are relatively scarce, for which the flow dispersion mechanisms and internal forces are not exactly the same as the ones in the submillimeter scale. The most representative millimeter-scale microreactor is the Advanced Flow Reactor (AFR) of Corning Inc., the relatively large characteristic sizes of which significantly increase its processing capacity [19,20,21,22]. Nevertheless, with the increase in flow rate, the stagnation zones in the AFR expand obviously, which makes the velocity distribution uneven and leads to the formation of local hot spots [23].

The prediction of flow pattern and its transformation is important in the field of microreactors. The flow pattern study can help us to understand the control force in the flow, thereby controlling its mass transfer process. The flow patterns of liquid–liquid systems are often affected by many factors, such as physical properties of the fluid, channel structures, operating conditions, etc. Therefore, it is difficult to propose a universal flow pattern map or its transition criteria [24]. Wang et al. [25] have studied flow patterns of liquid systems in different microchannels and found that the flow pattern diagrams varied for different dispersion systems and microchannels with complex structures. Therefore, for specific geometric structures and flow systems, analysis should be performed individually to obtain a more reliable flow pattern map. In this regard, research on two-phase flow has often been carried out in regular channels, such as circular or square channels [24,26,27], while the studies of flow pattern in complex channels with changing structures and characteristic sizes have been rarely reported.

Based on the split and recombine principle and the theory of chaotic convection, a butterfly-shaped microreactor with a characteristic size at the millimeter scale was proposed. The velocity fields, streamlines, and pressure fields of single-phase flow in the microreactor were obtained by a computational fluid dynamics (CFD) simulation, and the effect of flow rate on homogeneous mixing was quantified. The flow patterns and mass transfer behaviors in the microreactor were investigated by visualization techniques [28,29] through the standard extraction system of “n-butanol/succinic acid/water” [30], from which schematic diagrams of various flow patterns were given and a flow pattern map was established for regulating the flow behavior via controlling the flow rates of the two-phase flow. Moreover, the dependence between the flow pattern and mass transfer was discussed and the empirical relationship between volumetric mass transfer coefficient and Reynold number was established with a prediction error less than 20%.

## 2. Materials and Methods

### 2.1. Modeling Theory and Method

#### 2.1.1. Governing Equations

We studied incompressible Newton fluid at the microscale, which is always in the form of laminar flow, and the change in temperature was not considered. This analysis mainly involved the mass conservation equation and the momentum conservation equation.

The conservation of mass of the flow system was manifested as the continuity of fluid flow in the flow field. For each phase in the flow, the flow Constraint Equation (1) and volume fraction Constraint Equation (2) must be satisfied:

(1) $$\frac{\partial \left(\alpha_{i}\rho_{i}\right)}{\partial t}+\nabla •\left(\alpha_{i}\rho_{i}\vec{u_{i}}\right)=0$$

(2) $$\overset{n}{\underset{i=1}{\sum }}\alpha_{i}=1$$

In the equation, $\alpha_{i}$ and $\rho_{i}$ are the volume fraction and density of the phase, respectively. In the microreaction device, the flow state of the microfluid was a continuous flow without slip boundary conditions, so the conventional macroscopic Navier–Stokes Equation (3) could be used:

(3) $$\rho \frac{\partial \vec{u}}{\partial t}+\rho \vec{u}\cdot \nabla \vec{u}=-\nabla P+\mu \nabla^{2}\vec{u}+\rho \vec{g}+\vec{F}$$

In the equation, P is the pressure, $\vec{g}$ is the gravity coefficient, μ is the viscosity, and $\vec{F}$ is the external force on the unit volume fluid, which is also known as the source term. In the simulation process, the volume-weight-mixing-law was used to calculate the density, and the mass-weight-mixing-law was used to calculate the viscosity. The flow in the microchannel was mainly laminar, so the free diffusion of molecules played a dominant role in the mass transfer process. At a certain temperature, the diffusion caused by the thermal motion of molecules can be expressed by Fick’s Law Equation (4):

(4) $$J_{i}=-D\frac{dc_{i}}{dx}$$

In the equation, $c_{i}$ is the concentration of the component and D is the diffusion coefficient. The convection between the phases should also be taken into account in the material transfer process when the flow velocity increases or when local turbulence is produced due to the change in the channel geometry. Therefore, the convection–diffusion equation can be obtained as shown in Equation (5):

(5) $$\frac{\partial c_{i}}{\partial t}+\vec{u_{i}}\cdot \nabla c_{i}=D\cdot \nabla^{2}c_{i}$$

#### 2.1.2. Numerical Approach

Based on the design principles of split-and-recombine microreactors, a butterfly microreactor composed of serpentine channels was proposed in this research. During the flow process, the two-phase fluid flowed into the microreactor from different inlets and was divided into two by the butterfly shaped splitter plate in the middle. The velocity gradient was created through a curved channel on both sides, and the mixing was reinforced by internal obstacles. Then, the two streams converged into one and entered the next mixing unit. The fluid was repeatedly stretched, rotated, and sheared through multiple mixing units to promote mixing and mass transfer between phases.

Over the course of this study, a microreactor consisting of 6 cascade units was used, and each unit had a liquid-holding capacity of 120 μL. The liquid-holding capacity requirement could be easily achieved by increasing or decreasing the number of units.

**Figure 1:**
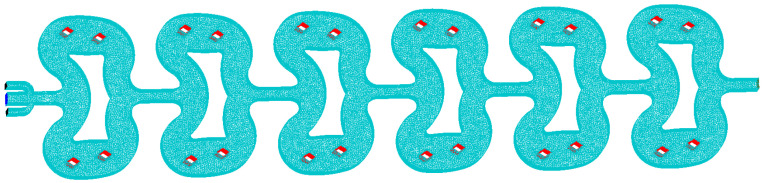
3D mesh of the microreactor composed of 4,278,327 elements.

In this study, ICEM CFD was used to construct the grid. The computational domain contained a total of 4,278,327 three-dimensional grid elements, as shown in Figure 1. For the inlet, outlet, and quadrangular prism shaped posts, we used a smaller mesh size to ensure the accuracy of the results.

A homogeneous simulation used water and ethanol as the flow phase, both of which used the same volumetric flow rates. The total flow rate ranged from 10–40 mL/min with an interval of 10 mL/min. The pressure–velocity coupling scheme was resolved by the SIMPLE (semi-implicit method for pressure-linked equations) algorithm, and the spatial discretization scheme adopted the second-order upwind style. The under-relaxation factors of pressure and momentum were 0.3 and 0.7, respectively. The steady-state calculation was used, and the residual was set to 1 × 10^−6^.

For homogeneous systems, related studies [31] proposed using the mixing index as an indicator of the degree of mixing, which was similar to the variance in the component concentration on the cross-section of the microchannel. The equation is shown in (6):

(6) $$M=1-\sqrt{\frac{1}{n}\overset{n}{\underset{i=1}{\sum }}\left(\frac{C_{i}-C_{0}}{C_{0}}\right)}$$

where M is defined as the mixing index of a certain cross-section of the channel; the closer the value is to 1, the more complete the mixing, and vice versa. n is the number of sampling points on the cross-section, $C_{i}$ is the concentration of components at each sampling point, and $C_{0}$ is the average concentration of the components on the cross-section.

### 2.2. Experimental Setup

#### 2.2.1. Fabrication

**Figure 2:**
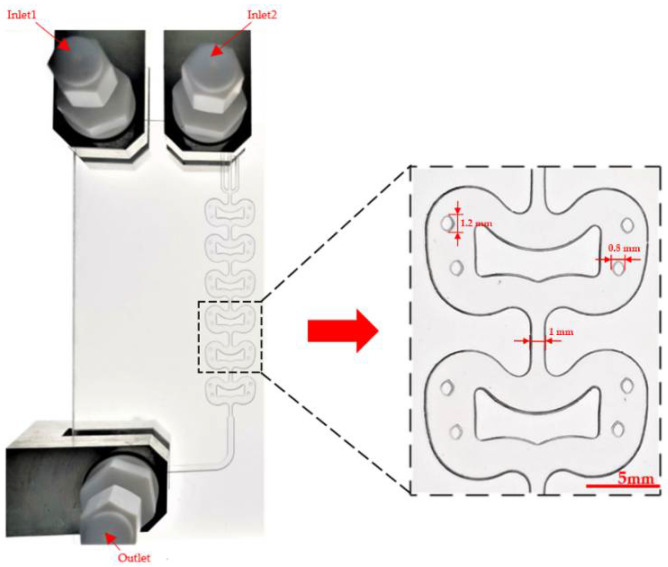
Image of the microreactor and the region of interest.

The image of the microreactor is shown in Figure 2. The microreactor was made of glass through femtosecond laser micromachining. This technology uses high-energy laser pulses to directly engrave microchannels in the interior of the high borosilicate glass, which has higher machining accuracy and avoids poor sealing. The external thickness of the microreactor is 5 mm, and the depth of the internal microchannel is 1.2 mm. It has two inlets and one outlet. The mixing part between the inlets and outlet consists of six butterfly-shaped units with a width of 15 mm and a height of 8.8 mm. A butterfly obstacle is located in the middle of each unit, and two pairs of quadrangular prism-shaped posts were located on both sides. The width of the butterfly obstacle was 8 mm, and the size of the post was 1.2 mm × 0.8 mm. The total internal volume of the microreactor was about 0.75 mL, with each butterfly unit having a volume of 120 μL. It should be noted that the microreactor was only used for preliminary study of flow and mass transfer behavior, and the total volume and mixing performance can be facilely improved by increasing the number of mixing units.

#### 2.2.2. Visualization System

In this study, a visualization system was set up to study the two-phase flow behavior in the microchannel, including a digital camera (Canon EOS 6D, Shimomaruko, Japan), a CCD industrial camera (Aosvi HK830, Guangzhou, China), a microscope lens, an LED ring light source, and an adjustable lifting arm. The dimensions are measured by using the built-in image measuring software of the industrial camera. It was calibrated by a standard ruler before measurement.

The visualization experiment used water and n-butanol as the flow phase, and the physical properties of both are shown in Table 1. During the experiment, inlet 1 was the water phase inlet, and inlet 2 was the organic phase inlet. The water phase flowed in from inlet 1 and split into two strands to enter the microreactor from both sides. The intermediate mixing units were selected as the region of interest (ROI) to eliminate the influence of the inlet effect and the outlet disturbance on the two-phase flow pattern. In the experiment, in order to obtain clearer images, Sudan III (Aladdin, 99.5%) was added to the organic phase as a dye, and no dye was added to the water phase.

#### 2.2.3. Extraction Experiment

This study used a standard low interfacial tension extraction system of n-butanol/succinic acid/water to quantify the two-phase mass transfer in the flow process. This system has been widely used in many studies [30,32], and the transfer species was succinic acid (Aladdin, 99.5%), which was transferred from the n-butanol phase (Macklin, 99.8%) to the water phase (deionized water) during the flow process. The total flow rate of the experiment ranged from 1–48 mL/min, and the influence of different two-phase volume ratios q (the volume flow ratio of the water phase to the n-butanol phase) on the extraction efficiency and volumetric mass transfer coefficient were investigated. A glass pipette with an inner diameter of 5 mm was used to separate the outlet stream of the microreactor. The concentration of succinic acid in the water phase was obtained by titration with 0.1 mol/L NaOH solution, and the extraction efficiency *E* and the volumetric mass transfer coefficient *K_L_a* were further calculated [32]. The equation for the extraction coefficient *E* is shown in (7):

(7) $$E=\frac{\left(C_{w,i}-C_{w,0}\right)}{\left(C_{w,i}-C_{w}^{*}\right)}$$

where $C_{w,i}$ and $C_{w,0}$ are the concentration of succinic acid at the inlet (which was zero for our experimental conditions) and outlet of the water phase, respectively, and $C_{w}^{*}$ is the equilibrium concentration of succinic acid in the water phase, which was defined by the partition coefficient m between the two phases and the volume flow ratio *q* ($q=\frac{Q_{w}}{Q_{0}}$) of the two phases. The equation is shown in (8):

(8) $$C_{w}^{*}=mC_{0}^{*}=\frac{mC_{0,i}}{mq+1}$$

where $C_{0}^{*}$ and $C_{0,i}$ are the equilibrium concentration of succinic acid in the organic phase and the concentration of succinic acid at the inlet of the organic phase (which was 0.2 mol/L for our experimental conditions), respectively. The equation of the volumetric mass transfer coefficient is shown in Equation (9):

(9) $$K_{L}a=\frac{1}{\tau }ln\left(\frac{c_{w}^{*}-c_{w,i}}{c_{w}^{*}-c_{w,o}}\right)$$

where τ is the residence time, which can be calculated from the two-phase flow rate and the volume of the microreactor.

## 3. Results and Discussion

In this section, the simulation results were postprocessed by CFD-POST, and the velocity fields, streamlines, and pressure fields in the microreactor were obtained. Subsequently, the flow and mass transfer behaviors under different flow conditions were investigated through the visualization systems and extraction experiments.

### 3.1. Computational Fluid Dynamics (CFD) Simulation Results

#### 3.1.1. Velocity Fields, Streamlines and Pressure Fields

**Figure 3:**
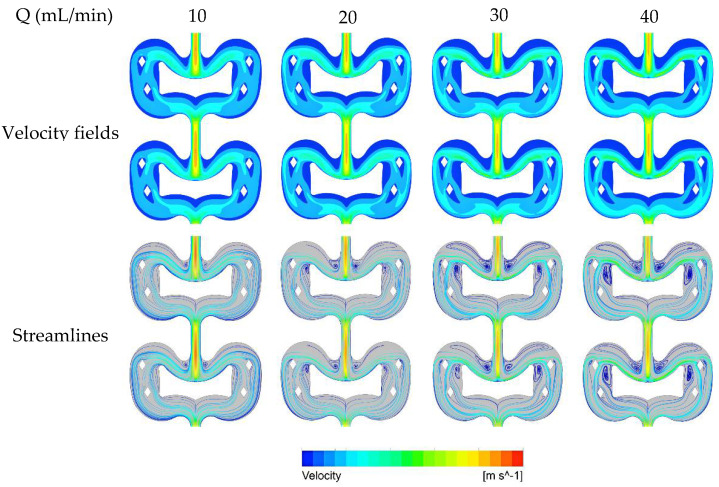
The velocity fields and streamlines of single-phase flow in the butterfly-shaped microreactor under different total flow rates (the closer the color is to blue, the slower the speed, and the closer the color is to red, the faster the speed).

The velocity fields and streamlines in the butterfly-shaped microreactor under different total flow rates are shown in Figure 3. Firstly, the fluid enters the mixing unit and splits into two streams by the butterfly obstacle, followed by a further split by four quadrangular prism-shaped posts. Then, the two streams re-converge and flow into the next mixing unit.

As shown in the velocity fields, the velocity distribution in each unit is relatively uniform, possibly due to the smooth curve structure. In each mixing unit, only a few stagnation zones were observed on both sides of the entrance and at the edges of lower part of the butterfly obstacle. The velocity distribution in each unit is identical, which indicates that the flow becomes fully developed within a short distance after entering the microreactor. The streamlines show that the size of the swirling zones expands with the increase of flow velocity, and the swirling intensity also increases with the flow velocity.

**Figure 4:**
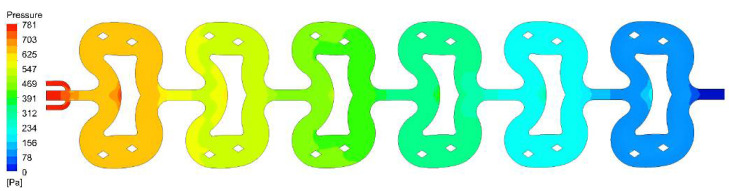
The pressure field in the butterfly-shaped microreactor at a total flow rate of 20 mL/min.

For a total flow rate range of 10–40 mL/min, the Reynolds number in the microreactor ranges from 126–504, indicating the flow is laminar. Therefore, the pressure drop follows the Hagen–Poiseuille equation [33]. Figure 4 shows the pressure field at a flow rate of 20 mL/min. Generally, the pressure distribution in each unit is uniform. There is a relatively larger pressure gradient at the inlet and outlet of each unit and the upper edges of each butterfly obstacle, due to the difference in flow rate distribution. For other flow rates, a similar type of pressure field was also observed.

#### 3.1.2. Homogeneous Mixing Efficiency

**Figure 5:**
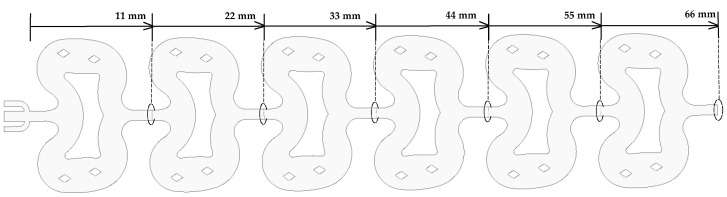
Schematic diagram of the position of each section.

**Figure 6:**
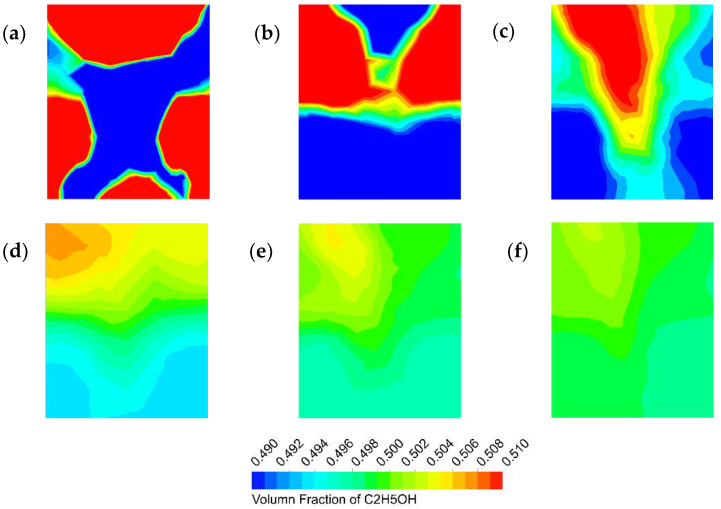
Contours of the ethanol volume fraction at cross-sections with different flow distances (40 mL/min): (**a**) 11 mm; (**b**) 22 mm; (**c**) 33 mm; (**d**) 44 mm; (**e**) 55 mm; and (**f**) 66 mm.

To investigate the variation in the mixing degree of the two-phase fluid along the flow distance, a series of cross-sections of the connecting pipe section between two adjacent mixing units of the microreactor was analyzed. The spacing between each section was 11 mm, and the volume fraction of ethanol in each section is shown in Figure 5 and Figure 6. The volume fraction of ethanol should be 0.5 when fully mixed since we used equal volume flow rate feeds for the two phases in the simulation. Blue represents the water phase, and red represents the ethanol phase. In the simulation, we used the feed method in which water was added from both sides of the inlet, and ethanol was added from the middle inlet. As shown in the figure, in the first section, the ethanol phase, which should be in the middle, was dispersed near the wall, while the water phase showed the opposite condition. The position alternation in the flow process was caused by the difference in velocity caused by the curve structure and the internal obstacle, which was favorable for mixing in the state of laminar flow. Overall, the two-phase flow demonstrated stratified flow at the beginning and then mixed flow due to splitting and recombining in the butterfly microreactor to form a homogeneous flow; the state of uniform mixing was basically reached at the fourth unit.

**Figure 7:**
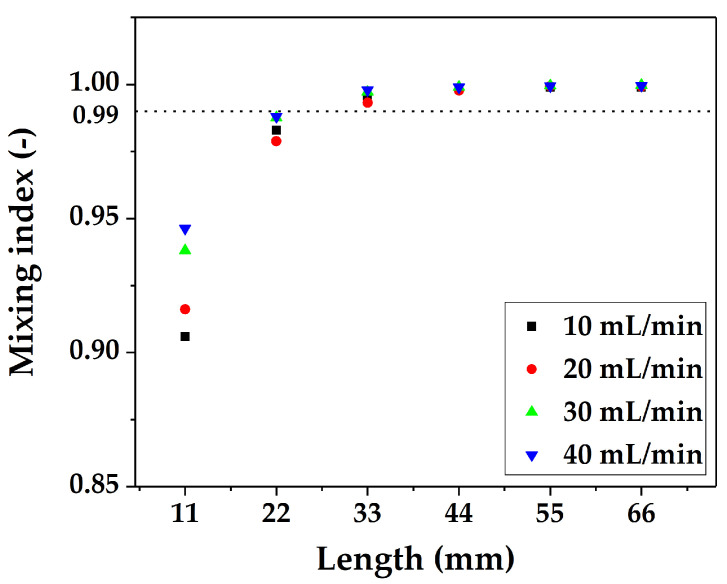
Variation in the mixing index with flow distance under different flow rates.

This study quantified the mixing degree based on the mixing index M. We uniformly selected 30 data points on the above cross-section and then used Equation (6) to obtain the change in the mixing index along the flow distance. Figure 7 shows the variation in the mixing index with the flow distance under different flow rates. The influence of the flow rate on the mixing index was mainly reflected in the short flow distance. As the flow rate increased, the intensity of convection between the fluids increased, so the mixing effect gradually increased. When a larger flow rate is used, the distance and time to reach complete mixing are both shorter. When the two-phase fluid arrived at the inlet of the fourth mixing unit, the mixing of the two-phase fluids reached 99%, which could basically be regarded as complete mixing.

### 3.2. Visualization Experiment Results

#### 3.2.1. Flow Pattern Diagrams

**Figure 8:**
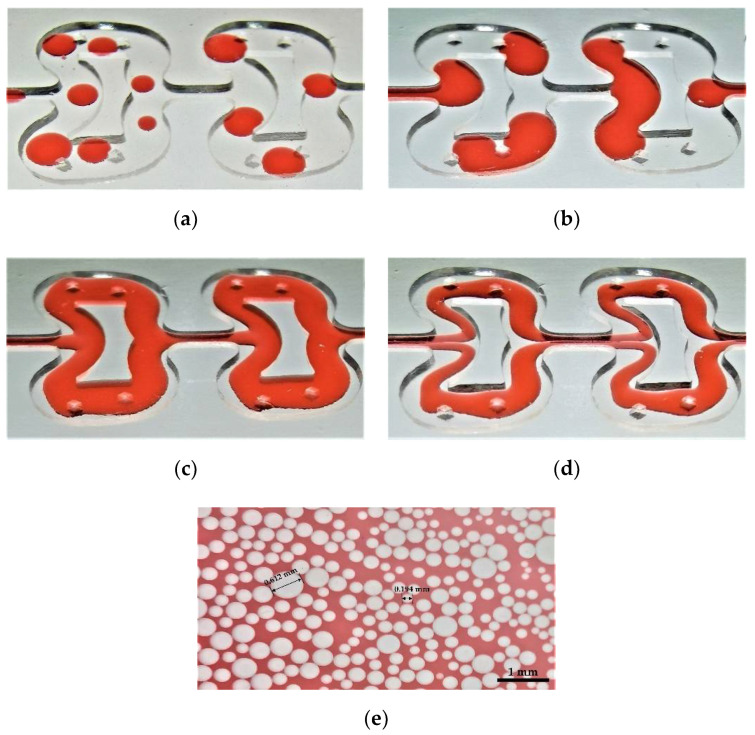
Experimental flow patterns in the microreactor: (**a**) droplet flow; (**b**) Taylor flow; (**c**) stratified flow; (**d**) annular flow; (**e**) dispersed flow.

The flow patterns in the microreactor were investigated by using the above visualization system. Five flow patterns, including droplet flow, Taylor flow, stratified flow, annular flow, and dispersed flow, were obtained at different apparent flow rates (*Re* = 8.3–1526), as shown in Figure 8. With the increase in the continuous phase flow rate, the ratio of continuous phase shear force to interfacial tension increases, which leads to the formation of the droplet flow and Taylor flow. With the increase in dispersed phase flow rate, the ratio of dispersed phase viscous force to interfacial tension increases, which restrains the shrinkage and fracture of the phase interface, thereby forming a stratified flow.

Compared with stratified flow, due to the increase in continuous phase flow, annular flow shown that the organic phase no longer flowed completely along the inner wall surface but was in the middle of the microchannel and formed a symmetrical flow pattern. Because the flow velocity of the dispersed flow was too fast, it was difficult to directly capture in the microreactor, so we collected the mixed liquid phase in a watch glass for observation.

#### 3.2.2. Flow Pattern Map

**Figure 9:**
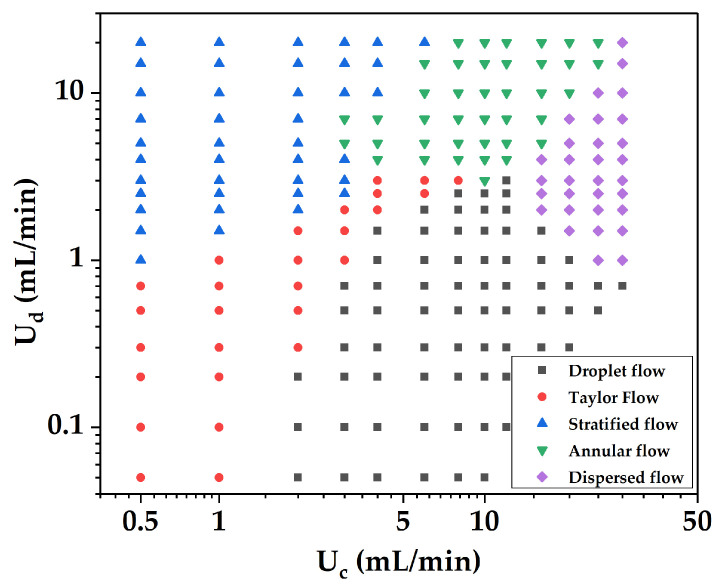
Flow pattern map of two-phase flow in the microreactor.

Based on the experimental results, the flow pattern map of the two phases in the microreactor was established, as shown in Figure 9, to predict the flow patterns under given operating conditions. The x and y axes are the continuous phase (water phase) flow rate and dispersed phase (organic phase) flow rate, respectively, and the region between different flow patterns is the transition boundary. The flow pattern map shows that under the constant flow rate of the continuous phase, the flow pattern gradually transitioned from droplet flow to Taylor flow and stratified/annular flow with the increase in the flow rate of the dispersed phase. The dispersed phase was sheared into countless small droplets under a higher flow rate of the two phases and evenly dispersed in the whole channel to form dispersed flow. The two phases were in full contact, and the volumetric mass transfer coefficient was very high.

In the microreactor, the two-phase flow pattern was mainly affected by the interfacial tension and shear force of the continuous phase. When the volume ratio of water to n-butanol *q* ≤ 0.5, stratified flow and annular flow easily formed; when *q* > 3, droplet flow and dispersed flow easily formed; and when *q* = 0.5–3, Taylor and stratified flow/annular flow easily formed.

#### 3.2.3. Flow Regimes of Droplet

The droplet flow is an ideal flow pattern. A single droplet can form a micro-reaction system, with excellent transfer performance and maneuverability [34,35]. Many studies have reported the applications of droplet or droplet-based microfluids, such as the preparation of nanomaterials [36], drug encapsulation [37] and bioanalysis [38]. The transfer performance of droplets depends on its size and monodispersity to a great extent. Therefore, the droplet flow in the microreactor was further investigated in this study. It should be noted that the data collection zones in this section are the regions of interest shown in Figure 2, that is, the two mixing units in the middle, rather than the entire microchannel. Table 2 shows the effect of the total flow rate on the number and average size of droplets at a fixed volume ratio of water to n-butanol (*q* = 4). It can be seen from the data that with the increase in the two-phase flow rate, the number of droplets in the ROI increases gradually, while the size of droplets decreases gradually. This is due to the increase in the ratio of the continuous phase shear force to the surface tension, which leads to the decrease of droplet size.

Table 3 shows the effect of the volume ratio of water to n-butanol on the number and average size of droplets at a fixed total flow rate of 8 mL/min. We can find that the number and size of droplets gradually decrease with the increase in *q*. When the volume ratio of water to n-butanol reduces by 75%, the number and size of droplets increase by 30% and 70%, respectively.

**Figure 10:**
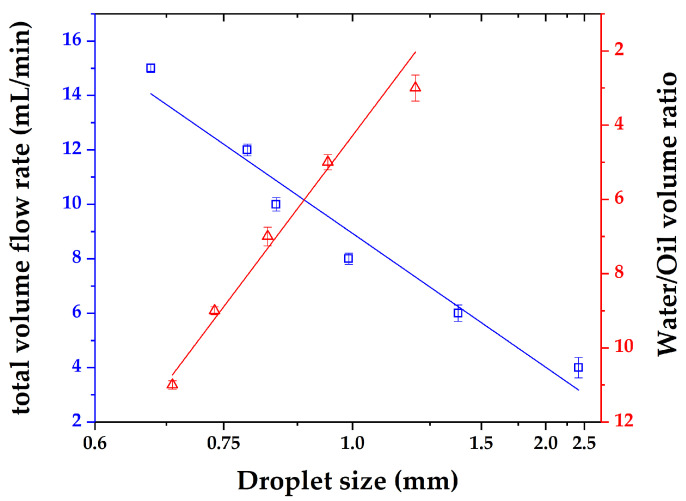
Relationship of droplet size to total flow rate (**blue**) and volume ratio of water to n-butanol (**red**).

The droplet size varies almost linearly with the total flow rate (at a fixed volume ratio of water to n-butanol) or the volume ratio of water to n-butanol (at a fixed total flow rate), as illustrated in Figure 10. At the same time, with the increase in the continuous phase flow rate, the standard deviation of droplet size decreases gradually, that is, the droplet size tends to be stable.

### 3.3. Extraction Experimental Results

#### 3.3.1. Extraction Efficiency and Volumetric Mass Transfer Coefficient

**Figure 11:**
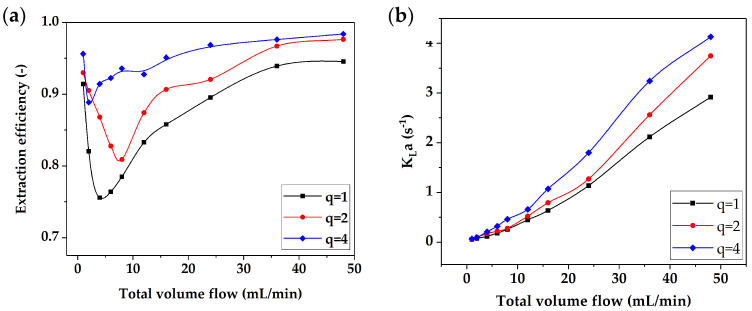
(**a**) Effect of flow rate on the extraction efficiency *E* and (**b**) volumetric mass transfer coefficient *K_L_a*.

The mass transfer behavior of two-phase flow in the microreactor was analyzed by using the standard low surface tension system “*n-butanol/succinic acid/water*”. In the experiment, the variation in extraction efficiency and the volumetric mass transfer coefficient with the total flow rate under different water/oil flow ratios was investigated. The results are shown in Figure 11.

Figure 11a shows that the extraction efficiency first decreased and then rose with increasing total flow rate under a fixed water/oil flow ratio, which was closely related to the two-phase flow pattern transition in the microreactor. When the total flow rate was in a low range, the two phases presented Taylor flow or stratified flow, the contact area of the two phases was basically unchanged, and the mass transfer between the two phases was mainly controlled by the residence time. Therefore, the extraction efficiency tended to decrease gradually. When the total flow rate increased, the flow shear force increased and made the flow pattern gradually transition to dispersed flow. At this time, the mass transfer was mainly controlled by the two-phase dispersion state. The higher the degree of dispersion, the higher the extraction efficiency. When the total flow was further increased, the two-phase flow pattern was dispersed flow, the mass transfer area was very large, and the extraction efficiency tended to a fixed value. At the same time, as the volume ratio of water to n-butanol increased, that is, as more water was used as the extractant, the degree of decrease in extraction efficiency gradually weakened.

Figure 11b shows that the volumetric mass transfer coefficient increased with the increasing flow rate. With the increase in flow velocity in the microchannel, the area of two-phase mass transfer and the rate of surface renewal increased, which made the volumetric mass transfer coefficient increase. At low flow rates, the effect of the volume ratio of water to n-butanol on the volumetric mass transfer coefficient was not obvious because of the long residence time of the two phases in the microreactor.

#### 3.3.2. Empirical Equation of Volumetric Mass Transfer Coefficient

Based on the complexity of the two-phase flow and mass transfer process, an empirical equation for predicting the volumetric mass transfer coefficient was derived by changing the total flow rate and volume ratio of two-phase flow in this study. The factors in the mass transfer process are transformed into a dimensionless number, as shown in Equation (10):

(10) $$R_{e_{q}}={\left(\frac{du_{m}\rho_{m}}{\mu_{m}}\right)}^{\frac{1}{4}}$$

and:

(11) $$\rho_{m}=\frac{q}{1+q}\rho_{w}+\frac{1}{1+q}\rho_{0}$$

(12) $$\mu_{m}=\frac{q}{1+q}\mu_{w}+\frac{1}{1+q}\mu_{0}$$

where d is the hydraulic diameter at the inlet; $u_{m}$ is the average flow velocity at the inlet; $\rho_{w}$, $\rho_{0}$ and $\rho_{m}$ are the water phase density, organic phase density and average density, respectively; and $\mu_{w}$, $\mu_{0}$ and $\mu_{m}$ are the water phase viscosity, organic phase viscosity, and average viscosity, respectively. The data point collection range was as follows: the total flow rate ranged from 1–48 mL/min, and the volume ratio of water to n-butanol ranged from 1–4. The following empirical equation can be obtained through Correlation (13):

(13) $$K_{L}a=1.04\times 10^{-3}{Re_{q}}^{5.416}+0.03523$$

**Figure 12:**
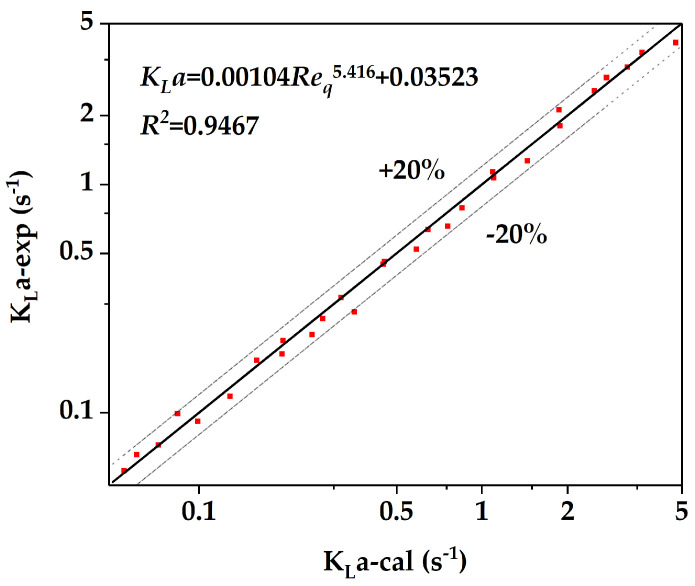
Comparison of the experimental value (**point**) and calculated fitting curve of volumetric mass transfer coefficient over Reynold number.

The experimental data of the volumetric mass transfer coefficient were compared with the calculated values of the empirical equation. The result is shown in Figure 12. The prediction error of the empirical correlation is within ±20%.

#### 3.3.3. Mass Transfer Performance Comparison

The volumetric mass transfer coefficient *K_L_a* can reflect the mixing capacity of the reactor. A number of studies [39,40,41] have been performed to investigate the *K_L_a* of various liquid–liquid contact systems. Table 4 compares the *K_L_a* of some conventional industrial systems, microreactors, and butterfly microreactors proposed in this study. The results showed that the *K_L_a* of the butterfly microreactor presented in this paper is 1~2 orders of magnitude higher than that of traditional industrial equipment and is equivalent to various types of microreactors.

## 4. Conclusions

The current study proposed a butterfly-shaped cascade microreactor with a characteristic size at the millimeter scale, the flow and mass transfer behaviors of which were investigated. The velocity fields, streamlines and pressure fields in the microreactor were obtained using CFD simulations. The stagnation area and swirling intensity increase slightly with the increase in the total flow rates, but the velocity and pressure distribution in each mixing unit is relatively uniform. The influence of flow rate on the homogeneous mixing efficiency was quantified by mixing index, which showed the mixing performance improves with increasing flow rates.

The flow behaviors in the microreactor were investigated using water and n-butanol as the flow medium, from which the schematic diagrams of various flow patterns were given and a flow pattern map for regulating the flow behavior via control of two-phase flow rates was also established. The results showed that when the water/butanol volume ratio surpassed 3, droplet flow easily formed; when the ratio was smaller than 0.5, stratified flow or annular flow easily formed; and when the ratio was between 0.5 and 3, Taylor flow dominated. The behavior of droplet flow in the microreactor was further investigated, which showed the number of droplets increased with the increasing flow rates of dispersed phase while the size of the droplets and its standard deviation decreased with the increasing flow rate of continuous phase.

The mass transfer behavior of two-phase flow in the microreactor was evaluated using the standard low surface tension system of “*n-butanol/succinic acid/water*”, and the effects of flow rates of the two phases on the extraction efficiency (*E*) and the volumetric mass transfer coefficient (*K_L_a*) were investigated. The results showed the dependence between flow pattern and mass transfer: *E* and *K_L_a* decreased when the flow pattern changed from the droplet flow to stratified flow/annular flow; when the flow pattern changed from the stratified flow/annular flow to dispersed flow, *E* and *K_L_a* were improved significantly. Furthermore, the empirical relationship between *K_L_a* and Reynold number was established with a prediction error less than 20%. The *K_L_a* of the butterfly microreactor presented in this paper is 1~2 orders of magnitude higher than that of traditional industrial liquid–liquid contactors and is equivalent to various types of microreactors.

## Figures and Tables

**Table 1:** Fluid properties of water and *n*-butanol at 20 °C.

Fluid	Density (kg/m^3^)	Viscosity (Pa·s)	Interfacial Tension (N/m)
Water	998	0.00100	0.0017
n-Butanol	810	0.00295	

**Table 2:** Droplet statistics for different total flow rates at *q* = 4.

Total Flow Rate (mL/min)	No. of Droplets	Average Droplet Size (mm)	Standard Deviation (mm)
4	9	2.407	0.376
6	14	1.376	0.300
8	18	0.990	0.204
10	23	0.835	0.242
12	26	0.786	0.216
15	33	0.657	0.155

**Table 3:** Droplet statistics for different volume ratio of water to n-butanol at a total flow rate of 8 mL/min.

Volume Ratio of Water to n-Butanol	No. of Droplets	Average Droplet Size (mm)	Standard Deviation (mm)
3	24	1.194	0.351
5	23	0.940	0.207
7	21	0.820	0.250
9	19	0.737	0.105
11	18	0.682	0.114

**Table 4:** Comparison of the volumetric mass transfer coefficients in liquid−liquid contactors.

Contactor Type	Chemical System	*K_L_a* (s^−1^)
Spray column [40]	Water/acetic acid/benzene	0.00175–0.063
Packed columns [40]	Methyl isobutyl ketone/uranyl nitrate-water	0.0004–1.02
Static mixers [39]	Oxygen/nitrogen/water	0.1–2.5
Capillary microchannel [40]	N-butanol/succinic acid/water	0.02–0.32
Rectangular glass microreactors [41]	Toluene/trichloroacetic acid/water+NaOH	0.2–0.5
Corning AFR [32]	N-butanol/succinic acid/water	0.07–3.31
Present work	N-butanol/succinic acid/water	0.046–4.13

## Nomenclature

$\vec{u}$	velocity vector (m/s)
P	pressure (Pa)
$\vec{g}$	gravity vector (m/s^2^)
$\vec{F}$	external force (N)
$J_{i}$	diffusion flux (kg/m^2^·s^−^^1^)
D	diffusion coefficient(m^2^/s)
$c_{i}$	concentration of phase *i* (mol/L)
M	mixing index (−)
$C_{i}$	concentration of ethanol of sampling point (mol/L)
$C_{0}$	average concentration of ethanol of cross-section (mol/L)
E	extraction efficiency (−)
q	volume ratio of water to n-butanol (−)
m	partition coefficient (−)
*K_L_a*	volumetric mass transfer coefficient (s^−^^1^)
$C_{w,i}$	concentration of succinic acid in the water phase at the inlet (mol/L)
$C_{w,0}$	concentration of succinic acid in the water phase at the outlet (mol/L)
$C_{0,i}$	concentration of succinic acid in the organic phase at the inlet (mol/L)
$C_{w}^{*}$	equilibrium concentration of succinic acid in the water phase (mol/L)
$C_{0}^{*}$	equilibrium concentration of succinic acid in the organic phase (mol/L)
d	hydraulic diameter at the inlet (m)
Greek symbols	
$\alpha_{i}$	volume fraction of phase *i* (−)
$\rho_{i}$	density of phase *i* (kg/m^3^)
$\rho_{m}$	average density (kg/m^3^)
$\rho_{w}$	water phase density (kg/m^3^)
$\rho_{0}$	organic phase density (kg/m^3^)
μ	viscosity (Pa·s)
$\mu_{m}$	viscosity (Pa·s)
$\mu_{w}$	water phase viscosity (Pa·s)
$\mu_{0}$	organic phase viscosity (Pa·s)
τ	residence time (s)
Dimensionless numbers	
*Re*	Reynolds number (−)
